# Clinical Practice Guideline on Cognitive Assessments for the Early Detection of Cognitive Impairment in Primary Care: A report from the Alzheimer's Association

**DOI:** 10.1002/alz70861_108896

**Published:** 2025-12-23

**Authors:** Malavika Tampi, Dorene M. Rentz, Fred C Ko, Karen L. Bell, Sharon A Brangman, Maria C. Carrillo, Noelle L Fields, Brent P. Forester, Jennifer R. Gatchel, Dana C Jones, Ambar Kulshreshtha, Jennifer Loveless, Simin Mahinrad, Lauren Pilcher, Martin J. Sliwinski, Peter J. Snyder, Heather M Snyder, Eric G Tangalos

**Affiliations:** ^1^ Alzheimer's Association, Chicago, IL USA; ^2^ Brigham and Women's Hospital, Harvard Medical School, Boston, MA USA; ^3^ Icahn School Of Medicine at Mount Sinai, New York, NY USA; ^4^ Columbia University Irving Medical Center, New York, NY USA; ^5^ SUNY Upstate Medical University, Syracuse, NY USA; ^6^ Medical & Scientific Relations Division, Alzheimer's Association, Chicago, IL USA; ^7^ The University of Texas at Arlington, Arlington, TX USA; ^8^ Tufts University School of Medicine, Boston, MA USA; ^9^ Department of Psychiatry, Massachusetts General Hospital, Harvard Medical School, Boston, MA USA; ^10^ Department of Family Medicine, HopeHealth, Inc, Florence, SC USA; ^11^ Emory University School of Medicine, Atlanta, GA USA; ^12^ Contract Methodologist, Houston, TX USA; ^13^ Contract Methodologist, Chicago, IL USA; ^14^ Pennsylvania State University, State College, PA USA; ^15^ University of Rhode Island, Kingston, RI USA; ^16^ Division of Primary Care Internal Medicine, Geriatrics and Palliative Care, Mayo Clinic, Rochester, MN USA

## Abstract

**Background:**

Cognitive impairment often goes undetected by clinicians due to time constraints, limited expertise, and lack of standardized tools. Structured cognitive assessments improve detection accuracy compared to unstructured clinical judgment. In 2013, the Alzheimer’s Association published recommendations to help primary care clinicians incorporate cognitive assessments into the Medicare Annual Wellness Visit. Since then, advances in cognitive assessment tools, availability of disease‐modifying therapies, and shifting expectations around early diagnosis have highlighted the need for updated guidance. In response, the Alzheimer’s Association is developing an evidence‐based clinical practice guideline for cognitive testing in primary care, using the GRADE approach, informed by a systematic review of the best available evidence.

**Method:**

A multidisciplinary panel formulated a clinical question to inform the development of this guideline. A systematic review was conducted (search range: 1999‐2024) to evaluate the diagnostic accuracy of ten cognitive tests (5‐Cog, AD8, GPCOG, IQCODE, Mini‐Cog, MIS, MoCA, QDRS, RUDAS, and SLUMS) for the early detection of cognitive impairment (including MCI and dementia), in adults aged 55+ in ambulatory settings. The panel also narratively summarized data on emerging digital tools.

**Result:**

Forty‐one studies meeting eligibility criteria were included in the systematic review. Results were analyzed separately by test (including short‐IQCODE and short‐MoCA) and for primary care and for ambulatory clinic settings (e.g., neurology, memory disorder clinics), as well as for English and Spanish language, resulting in 21 separate analyses. Only three analyses, all related to MoCA, allowed for meta‐analysis. The panel will use the evidence to inform conditional recommendations for or against each specific test.

**Conclusion:**

By evaluating the diagnostic performance of selected tests, this guideline aims to support healthcare professionals in primary care settings in effective triaging of individuals who may benefit from further cognitive evaluation. To enhance real‐world applicability, considerations such as administration time, ease of scoring, and diagnostic accuracy ‐ along with appropriate cut‐offs by race/ethnicity, education, and language ‐ should be prioritized. This guideline will be regularly updated over time to reflect emerging evidence and the needs of diverse populations.

